# The use of technology in type 2 diabetes and prediabetes: a narrative review

**DOI:** 10.1007/s00125-024-06203-7

**Published:** 2024-06-29

**Authors:** Alexandros L. Liarakos, Jonathan Z. M. Lim, Lalantha Leelarathna, Emma G. Wilmot

**Affiliations:** 1 Department of Diabetes and Endocrinology, University Hospitals of Derby and Burton NHS Foundation Trust, Royal Derby Hospital, Derby, UK; 2 School of Medicine, Faculty of Medicine and Health Sciences, University of Nottingham, Nottingham, UK; 3 Diabetes, Endocrinology and Metabolism Centre, Manchester University NHS Foundation Trust, Manchester Royal Infirmary, Manchester, UK; 4 Department of Diabetes, Imperial College Healthcare NHS Trust, London, UK; 5 Faculty of Medicine, Department of Metabolism, Digestion and Reproduction, Imperial College London, London, UK

**Keywords:** Automated insulin delivery, Closed loop, Continuous glucose monitoring, Continuous subcutaneous insulin infusion, Diabetes technology, Insulin pump, Prediabetes, Review, Type 2 diabetes

## Abstract

The increasing incidence of type 2 diabetes, which represents 90% of diabetes cases globally, is a major public health concern. Improved glucose management reduces the risk of vascular complications and mortality; however, only a small proportion of the type 2 diabetes population have blood glucose levels within the recommended treatment targets. In recent years, diabetes technologies have revolutionised the care of people with type 1 diabetes, and it is becoming increasingly evident that people with type 2 diabetes can also benefit from these advances. In this review, we describe the current knowledge regarding the role of technologies for people living with type 2 diabetes and the evidence supporting their use in clinical practice. We conclude that continuous glucose monitoring systems deliver glycaemic benefits for individuals with type 2 diabetes, whether treated with insulin or non-insulin therapy; further data are required to evaluate the role of these systems in those with prediabetes (defined as impaired glucose tolerance and/or impaired fasting glucose and/or HbA_1c_ levels between 39 mmol/mol [5.7%] and 47 mmol/mol [6.4%]). The use of insulin pumps seems to be safe and effective in people with type 2 diabetes, especially in those with an HbA_1c_ significantly above target. Initial results from studies exploring the impact of closed-loop systems in type 2 diabetes are promising. We discuss directions for future research to fully understand the potential benefits of integrating evidence-based technology into care for people living with type 2 diabetes and prediabetes.

**Graphical Abstract:**

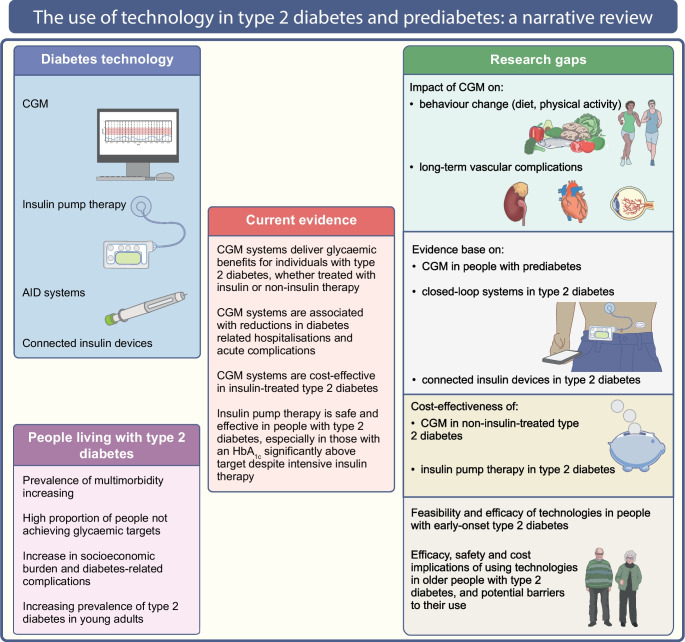

**Supplementary Information:**

The online version contains a slide of the figure for download available at 10.1007/s00125-024-06203-7.

## Introduction

Diabetes mellitus is a major public health issue characterised as a worldwide pandemic. A total of 537 million adults live with diabetes globally, with 90% of all cases diagnosed as type 2 diabetes [1]. This figure is predicted to rise by almost 50% in the next 20 years, which will be associated with increased rates of vascular complications [1]. Improved glucose management reduces the risk of vascular complications and mortality in people with type 2 diabetes [2–5]. However, data suggest that only around 50% of people with type 2 diabetes achieve the recommended HbA_1c_ target of <53 mmol/mol (7%) [6, 7], highlighting the need for better therapeutic options.

Technologies such as continuous glucose monitoring (CGM), insulin pumps and automated insulin delivery (AID) therapies have been shown to improve HbA_1c_, reduce hypoglycaemia and diabetes distress, and improve quality of life (QoL) in people with type 1 diabetes [8–10], and it is becoming increasingly evident that type 2 diabetes populations can also benefit from these advances [11, 12].

**Fig. 1:**
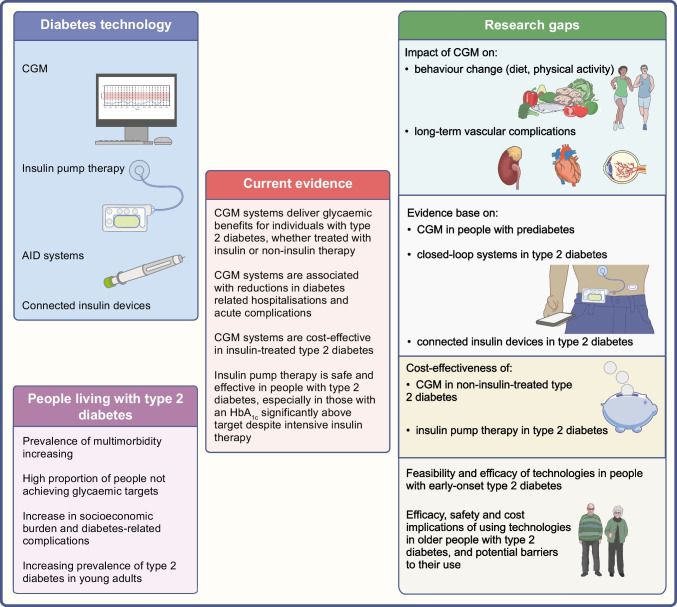
The use of technology in type 2 diabetes and prediabetes. This figure describes the current evidence and research gaps in the use of technology in type 2 diabetes and prediabetes. CGM improves glucose management in insulin- and non-insulin-treated type 2 diabetes, while the role of CGM in prediabetes requires further research. Insulin pumps improve glucose management in individuals with type 2 diabetes, especially in those with high HbA_1c_ despite intensive insulin therapy. The impact of CGM on behaviour changes and vascular complications, and the evidence base on connected insulin devices and closed-loop systems in type 2 diabetes, require further investigation. This figure is available as a downloadable slide

The aim of this review is to describe the current evidence regarding the role of technologies in people with type 2 diabetes, based on randomised trials, observational studies, systematic reviews and meta-analyses. We used the keywords ‘type 2 diabetes’, ‘diabetes technology’, ‘continuous glucose monitoring’, ‘flash glucose monitoring’, ‘intermittently-scanned continuous glucose monitoring’, ‘real-time continuous glucose monitoring’, ‘continuous subcutaneous insulin infusion’, ‘insulin pump’, ‘closed-loop’, ‘automated insulin delivery’, ‘artificial pancreas’, ‘connected insulin devices’, ‘smart insulin pen’ and ‘smart insulin pen caps’ alone and in combination to retrieve available literature from PubMed from inception until January 2024. The current evidence and research gaps in the use of technology in type 2 diabetes and prediabetes (defined as impaired glucose tolerance and/or impaired fasting glucose and/or HbA_1c_ levels between 39 mmol/mol [5.7%] and 47 mmol/mol [6.4%]) are illustrated in Fig. 1.

## CGM in type 2 diabetes

Current glucose monitoring technology enables intermittently scanned CGM (isCGM) and real-time CGM (rtCGM). isCGM involves sensors that need to be scanned to provide glucose values, while in rtCGM the sensors display glucose data on a reader or app automatically, without the need for scanning.

A meta-analysis of 26 RCTs (17 rtCGM, nine isCGM), involving 2783 people with type 2 diabetes, showed that, compared with self-monitoring of blood glucose (SMBG), rtCGM and isCGM reduced HbA_1c_ by 0.19 percentage points (pp) (2 mmol/mol) (95% CI −0.34, −0.04 pp) and 0.31 pp (3 mmol/mol) (95% CI −0.46, −0.17 pp), respectively. Time in range (TIR) increased significantly in isCGM users (three RCTs) and non-significantly in rtCGM users (six RCTs) [13]. CGM did not significantly impact glucose concentrations, glucose variability, measures of body composition, blood pressure or lipid levels [14, 15]. There was no difference in risk of hypoglycaemia between CGM and SMBG [14, 16–19]. Treatment satisfaction improved with CGM use, especially with newer generation systems, compared with SMBG [13, 17, 20, 21]. A more recent systematic review of CGM in adults with type 2 diabetes, which excluded studies investigating professional CGM and those combining CGM with additional glucose-lowering treatment, identified 12 RCTs (eight rtCGM, four isCGM) involving 1248 people [22]. Compared with SMBG, CGM (isCGM or rtCGM) resulted in a reduction in HbA_1c_ (mean difference [MD] −3.43 mmol/mol [−0.31 pp], 95% CI −4.75, −2.11 mmol/mol; *p*<0.00001). The effect size was comparable between studies including individuals on insulin ± oral therapy (MD −3.27 mmol/mol [−0.30 pp], 95% CI −6.22, −0.31 mmol/mol; *p*=0.03) and studies including those on oral therapy only (MD −3.22 mmol/mol [−0.29 pp], 95% CI −5.39, −1.05 mmol/mol; *p*=0.004). Using rtCGM showed a trend towards a larger effect (MD −3.95 mmol/mol [−0.36 pp], 95% CI −5.46, −2.44 mmol/mol; *p*<0.00001) than using isCGM (MD −1.79 mmol/mol [−0.16 pp], 95% CI −5.28, 1.69 mmol/mol; *p*=0.31). CGM compared with SMBG was also associated with increased TIR (+6.36%, 95% CI +2.48%, +10.24%; *p*=0.001) and decreased time below range (TBR) (−0.66 pp, 95% CI −1.21, −0.12 pp; *p*=0.02). No significant differences in severe hypoglycaemia or macrovascular complications were found between CGM and SMBG. No trials reported data on microvascular complications [22]. Table 1 summarises the main findings of the key RCTs on CGM use in type 2 diabetes.

**Table 1:** Evidence on the use of CGM in type 2 diabetes from key randomised trials

Study (first author, year, trial name)	Study details	Participant characteristics^a^	Medication use (%)^a^	Primary outcome and results
Aronson 2023, IMMEDIATE [11]	• Two-arm RCT • Intervention duration: 16 weeks • Study duration: 16 weeks • Intervention^b^ vs comparator: isCGM + DSME vs DSME	• No. of participants: 58/58 • Mean age: 59.2/57.6 years • Baseline HbA_1c_: 69/72 mmol/mol (8.5/8.7%)	Non-insulin-treated T2D • Metformin: 100/96 • SU: 55/43 • SGLT2i: 35/43 • DPP-4i: 43/47 • GLP-1RA: 28/35	• Primary outcome: % TIR in the final 2 week period • isCGM + DSME arm had a greater mean TIR by 9.9 pp (2.4 h) (95% CI −17.3, −2.5 pp; *p*<0.01) and lower TAR by 8.1 pp (1.9 h) (95% CI 0.5, 15.7 pp; *p*=0.037) than DSME group • isCGM + DSME arm had a greater reduction in mean HbA_1c_ by 0.3 pp (3 mmol/mol) (95% CI −0.7, 0 pp; *p*=0.048) than DSME arm • Glucose monitoring satisfaction was higher in the intervention group than the control group (MD +0.5, 95% CI +0.3, +0.7; *p*<0.01)
Ajjan 2023, LIBERATES [18]	• Two-arm RCT • Intervention duration: 12 weeks • Study duration: 12 weeks • Intervention^b^ vs comparator: isCGM vs SMBG	• No. of participants: 69/72 • Mean age: 62/63 years • Baseline HbA_1c_: 75/73 mmol/mol (9.0/8.8%)	• Insulin: 52.2/47.2 • SU: 47.8/52.8 • Metformin: 72.5/77.8 • DPP-4i: 21.7/15.3 • GLP1-RA: 7.2/6.9 • SGLT2i: 10.1/20.8 • Thiazolidinedione: 2.9/0.0	• Primary outcome: TIR on days 76–90 post randomisation • isCGM was associated with increased TIR by 17 min/day (95% credible interval −105, +153), with 59% probability of benefit • Lower hypoglycaemic exposure on days 76–90 (−80 min/day, 95% CI −118, −43) and days 16–30 (−28 min/day, 95% CI −92, 2) in isCGM users • Similar HbA_1c_ reduction (~7 mmol/mol [0.7 pp]) in isCGM and SMBG groups vs baseline • Glycaemic emergencies and mortality rates were not increased in isCGM users • QoL measures marginally favoured isCGM
Moon 2023 [29]	• Three-arm RCT • Intervention duration: 1–2 weeks • Study duration: 24 weeks • Interventions^b^ vs comparator: group 1 – one session of rtCGM at week 1; group 2 – two sessions of rtCGM at weeks 1 and 12; control group – SMBG	• No. of participants: 18/15/15 • Mean age: 55.6/53.9/50.7 years • Baseline HbA_1c_: 67/66/65 mmol/mol (8.3/8.2/8.1%)	Non-insulin-treated T2D • Metformin: 100/100/100 • SU: 66.7/73.3/40.0 • DPP-4i: 72.2/80.0/86.7 • SGLT2i: 44.4/26.7/13.3 • Thiazolidinedione: 38.9/40.0/66.7	• Primary outcome: change in HbA_1c_ at 6 months • At 6 months, only group 2 achieved significant HbA_1c_ reduction (adjusted difference −0.68 pp [–7 mmol/mol], 95 CI –1.23, –0.13 pp; *p*=0.018) vs control group • HbA_1c_ reduction was observed in group 1 (adjusted difference −0.60 pp [–6 mmol/mol], 95% CI –1.19, –0.02 pp; *p*=0.044) and group 2 (adjusted difference −0.64 pp [–6 mmol/mol], 95% CI –1.15, –0.14 pp; *p*=0.014) vs control group at 3 months
Choe 2022, PDF [12]	• Two-arm RCT • Intervention duration: 12 weeks • Study duration: 12 weeks • Intervention^b^ vs comparator: isCGM + structured education vs conventional diabetes care	• No. of participants: 63/63 • Mean age: 58.6/57.5 years • Baseline HbA_1c_: 63/63 mmol/mol (7.9/7.9%)	• Insulin: 32.8/22.6 • Number of non-insulin therapies: - 1: 13.8/12.9 - 2: 48.3/46.8 - 3: 34.5/38.7 - 4: 1.7/1.6	• Primary outcome: change in HbA_1c_ from baseline • isCGM was associated with greater improvement in HbA_1c_ than standard care (risk-adjusted difference −0.50 pp [–5 mmol/mol], 95% CI −0.74, −0.26 pp; *p*<0.001) • Greater reduction in fasting blood glucose (−0.9 mmol/l [–16.5 mg/dl], 95% CI –1.7, –0.2 mmol/l [–30, –3 mg/dl]; *p*=0.017) and body weight (−1.5 kg, 95% CI −2.7, −0.3; *p*=0.013) in intervention group • Diabetes Self-Care Activities Questionnaire score (Korean version) increased in both groups but to a greater extent in the intervention group (MD +4.8, 95% CI +1.7, +8.0; *p*=0.003) No severe hyperglycaemia/hypoglycaemia reported in either group
Martens 2021, MOBILE [14]	• Two-arm RCT • Intervention duration: 32 weeks • Study duration: 32 weeks • Intervention^b^ vs comparator: rtCGM vs SMBG	• No. of participants: 116/59 • Mean age: 56/59 years • Baseline HbA_1c_: 76/75 mmol/mol (9.1/9.0%)	Insulin: one or two daily injections of long- or intermediate-acting basal insulin without prandial insulin, with or without non-insulin glucose-lowering medications	• Primary outcome: HbA_1c_ at 8 months • Mean HbA_1c_ decreased by 1.1 pp [12 mmol/mol] (from 9.1% [76 mmol/mol] to 8.0% [64 mmol/mol]) in rtCGM group and by 0.6 pp [7 mmol/mol] (from 9.0% [75 mmol/mol] to 8.4% [68 mmol/mol]) in SMBG group (MD −0.4 pp [–5 mmol/mol], 95% CI −0.8, −0.1 pp; *p*=0.02) • TIR increased (adjusted difference +15 pp, 95% CI +8, 23; *p*<0.001), TAR (>13.9 mmol/l [>250 mg/dl]) decreased (adjusted difference −16 pp, 95% CI −21, −11; *p*<0.001) and hypoglycaemia (<3.9 mmol/l [<70 mg/dl]) decreased (adjusted difference −0.24 pp, 95% CI −0.42, −0.05; *p*=0.02) in rtCGM group vs SMBG group • Severe hypoglycaemic events were not increased in rtCGM group
Price 2021 [28]	• Two-arm RCT • Intervention duration: three sessions (baseline, week 4 and 8) • Study duration: 12 weeks • Intervention^b^ vs comparator: rtCGM vs SMBG	• No. of participants: 46/24 • Mean age: 59/61 years • Baseline HbA_1c_: 68.3/69.4 mmol/mol (8.4/8.5%)	Non-insulin-treated T2D • Treated with two or more non-insulin therapies	• Primary outcome: change in HbA_1c_ from baseline • No difference in mean HbA_1c_ reduction from baseline between rtCGM and SMBG groups (−0.5 pp [–5 mmol/mol] vs −0.3 pp [–3 mmol/mol]; *p*=0.74) at week 12 • 34.1% of rtCGM users vs 17.4% of SMBG users achieved a target HbA_1c_ <7.5% [<58 mmol/mol] (between-group difference *p*=0.12) • Mean TIR at week 8 vs baseline increased for rtCGM group (56.3% vs 63.1%) but decreased for SMBG group (68.4% vs 55.1%)
Cox 2020 [20]	• Two-arm RCT • Intervention duration: 8 weeks • Study duration: 24 weeks • Intervention^b^ vs comparator: rtCGM vs SMBG	• No. of participants: 20/10 • Mean age: 54/51 years • Baseline HbA_1c_: 74/73 mmol/mol (8.9/8.8%)	Non-insulin-treated T2D • No details on types of non-insulin glucose-lowering medications reported	• Primary outcome: change in HbA_1c_ • rtCGM was associated with reduction in HbA_1c_ (from 8.9% to 7.6% [from 74 to 60 mmol/mol]) vs reduction from 8.8% to 8.7% [from 73 to 72 mmol/mol] for SMBG (*p*=0.03) • rtCGM was associated with improved QoL (*p*=0.01) and diabetes knowledge (*p*=0.001) and reduced diabetes distress (*p*=0.02)
Wada 2020 [30]	• Two-arm RCT • Intervention duration: 12 weeks • Study duration: 24 weeks • Intervention^b^ vs comparator: isCGM vs SMBG	• No. of participants: 49/51 • Mean age: 58.1/58.7 years • Baseline HbA_1c_: 61.1/62.3 mmol/mol (7.83/7.85%)	Non-insulin-treated T2D • SU: 32.7/27.5 • Metformin: 69.4/62.7 • DPP-4i: 81.6/78.4 • SGLT2i: 42.9/37.3 • GLP-1 RA: 2.0/5.9 • Glinide: 20.4/21.6 • α-Glucosidase inhibitor: 26.5/35.3 • Pioglitazone: 8.2/13.7	• Primary outcome: change in HbA_1c_ • Mean HbA_1c_ decreased from baseline to 12 weeks in isCGM users (−0.43 pp [−4.7 mmol/mol]; *p*<0.001) and SMBG users (−0.30 pp [−3.3 mmol/mol]; *p*=0.001) • Mean HbA_1c_ decreased from baseline to 24 weeks in isCGM users but not in SMBG group (isCGM: −0.46 pp [−5.0 mmol/mol], *p*<0.001; SMBG: −0.17 pp [−1.8 mmol/mol], *p*=0.124; between-group difference: −0.29 pp [−3.2 mmol/mol], *p*=0.022) • DTSQ score improved in isCGM group vs SMBG group (difference in adjusted means +3.4, 95% CI +1.9, +5.0; *p*<0.001)
Ajjan 2019 [19]	• Three-arm RCT • Intervention duration: 24 weeks • Study duration: 24 weeks • Interventions^b^ vs comparator: group 1 – isCGM (two wears) + SMBG; group 2 – isCGM (four wears) + SMBG; control group – SMBG	• No. of participants: 46/50/52 • Mean age: 63.9/61.7/65.0 years • Baseline HbA_1c_: 71/71/71 mmol/mol (8.7/8.7/8.7%)	• Insulin (basal only): 52.2/42.0/40.4 • Insulin (basal–bolus): 41.3/50/50 • Insulin (biphasic): 6.5/8.0/9.6	• Primary outcome: TIR in group 2 comparing baseline with follow-up • In group 2, TIR was similar between baseline and follow-up (days 172–187) (15.0±5.0 h/day vs 14.1 ± 4.7 h/day; *p*=0.159) • HbA_1c_ decreased by 4.9 mmol/mol (0.44 pp) (*p*<0.001) from baseline to study end in group 2 • HbA_1c_ was lower in group 2 than control group at study end by 5.4 mmol/mol (0.48 pp) (*p*=0.004), without increased time in hypoglycaemia (*p*=0.178) • Treatment satisfaction scores improved in group 2 vs control group (*p*=0.023)
Yaron 2019 [23]	• Two-arm RCT • Intervention duration: 10 weeks • Study duration: 10 weeks • Intervention^b^ vs comparator: isCGM vs SMBG	• No. of participants: 53/48 • Mean age: 67.6/65.9 years • Baseline HbA_1c_: 71.4/67.7 mmol/mol (8.68/8.34%)	• Insulin (MDI): 100/100 • SU: 0.0/4.2 • Metformin: 71.7/72.9 • DPP-4i: 7.5/14.6 • SGLT2i: 24.5/27.7 • GLP-1 RA: 35.8/31.3	• Primary outcome: treatment satisfaction • Compared with SMBG group, isCGM group found the treatment significantly more flexible (*p*=0.019) and would recommend it to their counterparts (*p*=0.023) • HbA_1c_ decreased by 0.82 pp (9 mmol/mol) and 0.33 pp (3.6 mmol/mol) in the isCGM and SMBG groups, respectively (*p*=0.005)
Ilany 2018 [16]	• Two-arm RCT • Intervention duration: 16 weeks • Study duration: 24 weeks • Intervention^b^ vs comparator: isCGM + glulisine before a meal with the highest glucose elevation based on sensor data vs SMBG + pre-breakfast glulisine	• No. of participants: 60/61 • Mean age: 63/63 years • Baseline HbA_1c_: 68/69 mmol/mol (8.4/8.5%)	• Insulin: 100/100 (glargine: 65.0/68.3; detemir: 30.0/25.0; glulisine: 40.4/82.7)	• Primary outcome: HbA_1c_ at week 24 • No difference in HbA_1c_ reduction from baseline to follow-up between isCGM and SMBG groups (−0.54 pp [6 mmol/mol]; 95% CI −0.79, −0.3 pp vs −0.48 pp [5 mmol/mol]; 95% CI −0.76, –0.2 pp; *p*=0.75) • Frequency of hypoglycaemic events did not differ between isCGM and SMBG groups (52% vs 36%; *p*=0.08)
Beck 2017, DIAMOND [15]	• Two-arm RCT • Intervention duration: 24 weeks • Study duration: 24 weeks • Intervention^b^ vs comparator: rtCGM vs SMBG	• No. of participants: 79/79 • Mean age: 60/60 years • Baseline HbA_1c_: 69/69 mmol/mol (8.5/8.5%)	• Insulin (MDI): 100/100	• Primary outcome: change in HbA_1c_ at 24 weeks after randomisation • HbA_1c_ decreased to 7.7% (61 mmol/mol) in the rtCGM group and 8.0% (64 mmol/mol) in the control group at 24 weeks (adjusted difference in mean change −0.3 pp [−3 mmol/mol], 95% CI −0.5, 0.0 pp; *p*=0.022) • No difference in CGM-measured hypoglycaemia or QoL outcomes between rtCGM and SMBG groups
Haak 2017, REPLACE [17]	• Two-arm RCT • Intervention duration: 24 weeks • Study duration: 24 weeks • Intervention^b^ vs comparator: isCGM vs SMBG	• No. of participants: 149/75 • Mean age: 59.0/59.5 years • Baseline HbA_1c_: 72.0/73.5 mmol/mol (8.7/8.8%)	• Insulin (intensive insulin therapy): 100/100	• Primary outcome: difference in HbA_1c_ at 6 months • No difference in change in HbA_1c_ between isCGM and SMBG groups (−3.1 mmol/mol [–0.29 pp] vs −3.4 mmol/mol [–0.31 pp] ; *p*=0.822) • In people aged <65 years, rtCGM group had a greater improvement in HbA_1c_ than SMBG group (−5.7 mmol/mol [–0.53 pp] vs −2.2 mmol/mol [–0.2 pp]; *p*=0.03) • Time in hypoglycaemia (<3.9 mmol/l ([<70 mg/dl]) reduced by 43% (0.47 ± 0.13 h/day) (*p* <0.001) and time in hypoglycaemia (<3.1 mmol/l [<55 mg/dl]) reduced by 53% (0.22 ± 0.07 h/day) (*p*=0.0014) in isCGM group vs SMBG group • Treatment satisfaction was higher in isCGM group than SMBG group (DTSQ 13.1 ± 0.5 vs 9.0 ± 0.72; *p*<0.0001)
Tang 2014 [21]	• Two-arm RCT • Intervention duration: 24 weeks • Study duration: 24 weeks • Intervention^b^ vs comparator: rtCGM vs SMBG	• No. of participants: 40 in total • Mean age: 59/60 years • Baseline HbA_1c_: 68/73 mmol/mol (8.4/8.8%)	• Insulin alone or in combination with oral agent	• Primary outcome: treatment satisfaction • SMBG group reported higher overall treatment satisfaction than rtCGM users (DTSQ 33.41 vs 24.80; *p*<0.001)

^a^Data are presented for intervention/control or group 1/group 2/control, unless stated otherwise

^b^Type of CGM

*DPP-4i*, dipeptidyl peptidase 4 inhibitor; DSME, diabetes self-management education; DTSQ, diabetes treatment satisfaction questionnaire; GLP-1RA, glucagon-like peptide-1 receptor agonist; MDI, multiple daily insulin injections; pp, percentage points; SGLT2i, sodium-glucose co-transporter-2 inhibitor; SU, sulfonylurea; T2D, type 2 diabetes; TAR, time above range

### CGM use in people with type 2 diabetes on intensive insulin therapies

The DIAMOND RCT [15] showed that, compared with SMBG, rtCGM resulted in a greater HbA_1c_ reduction (MD −0.3 pp [–3 mmol/mol]) in a type 2 diabetes population treated with multiple daily insulin injections (MDI). However, the study did not incorporate structured diabetes education to optimise self-management and included people undertaking SMBG at least twice daily at baseline, while the control group were asked to perform SMBG four or more times daily. This may have resulted in underestimation of the impact of rtCGM on plasma glucose levels. In the REPLACE RCT, isCGM resulted in no difference in HbA_1c_ compared with SMBG. Nevertheless, the hypoglycaemia burden decreased and treatment satisfaction improved in isCGM users. An inclusion criterion of SMBG at least twice daily at baseline was reported and no education on data interpretation was provided [17], suggesting possible underestimation of the impact of isCGM on HbA_1c_. Another RCT of isCGM vs SMBG in a type 2 diabetes population on MDI showed that, although the primary outcome of treatment satisfaction was not met (*p*=0.053), users reported more flexibility (*p*=0.019) and would recommend isCGM to others (*p*=0.023) [23].

Overall, using CGM in those on intensive insulin therapy is beneficial. Several RCTs and real-world retrospective studies support CGM use, demonstrating improvements in HbA_1c_ and decreased frequency and severity of hypoglycaemia [24–27]. However, to date, no studies have investigated the impact of CGM in people with type 2 diabetes treated with mixed insulin; further research is required to evaluate the potential benefits in this group.

### CGM use in people with type 2 diabetes on basal insulin

The MOBILE RCT [14] found that, compared with SMBG, rtCGM resulted in a greater HbA_1c_ reduction (MD −4 mmol/mol [–0.4 pp]), improved TIR and decreased time above range (TAR) and TBR in a type 2 diabetes population treated with basal insulin (*p*<0.05 for all). The total dose of insulin and body weight did not differ between groups, which raises the possibility that rtCGM use may be directly associated with dietary and activity changes. This is an area that needs to be addressed in future research to gain a more detailed understanding of how CGM may drive glycaemic improvements in this group.

### CGM use in people with type 2 diabetes on non-insulin therapy

A pilot RCT of a structured diabetes education programme with episodic rtCGM use in a non-insulin-treated type 2 diabetes population demonstrated no significant HbA_1c_ improvement compared with SMBG [28], while an RCT of intermittent short-term use of rtCGM compared with SMBG found a 0.64 pp (6 mmol/mol) HbA_1c_ reduction (*p*=0.014) [29]. In another RCT [30], isCGM users showed a higher HbA_1c_ reduction than SMBG users at 24 weeks (MD –3.2 mmol/mol [−0.29 pp]; *p*=0.022). The IMMEDIATE RCT explored the glycaemic efficacy of isCGM plus diabetes self-management education compared with education alone in a type 2 diabetes population on at least one non-insulin therapy [11]. TIR at 4 months was higher in isCGM users (*p*=0.009), with little change in medication use (non-insulin glucose-lowering therapies were added for <10% of participants in each arm). This raises the possibility that CGM use may change behaviours, impacting glycaemic outcomes. The effect of CGM use on behaviour change is an area ripe for future research.

A retrospective analysis of 728 people with type 2 diabetes on non-insulin therapies using isCGM found a 1.6 pp (16 mmol/mol) HbA_1c_ reduction (*p*<0.001); a limitation of this analysis was the lack of a control group [31].

### CGM use and acute diabetes-related complications and hospitalisation

The RELIEF [32] retrospective study evaluated 40,846 people with type 2 diabetes (and 33,165 individuals with type 1 diabetes) in the first 12 months following isCGM initiation. Most within the type 2 diabetes cohort were treated with MDI, while a small proportion were treated with basal insulin or oral agents only. Twelve months following isCGM initiation, hospitalisation for acute diabetes complications decreased by 39% [32]. Specifically, in the type 2 diabetes population, the annual percentage of hospital admissions decreased for diabetic ketoacidosis (DKA) (from 1.7% to 0.82%), hypoglycaemia (from 0.7% to 0.62%), diabetes-related comas (from 0.23% to 0.16%) and hyperglycaemia (from 0.12% to 0.09%). The 2-year follow-up showed a persistent reduction in acute diabetes-related hospitalisations, from 2.0% before initiating isCGM to 0.75% at 1 year and 0.6% at 2 years follow-up [33]. Similarly, in a retrospective study carried out in the Netherlands, use of isCGM reduced diabetes-related hospital admissions from 13.7% to 4.7% (*p*<0.05) [34].

The LIBERATES RCT [18] investigated the effect of isCGM vs SMBG on blood glucose levels in a type 2 diabetes population with acute myocardial infarction, already treated with therapies that may result in hypoglycaemia. Although there was no significant difference in HbA_1c_ or TIR between groups, isCGM significantly reduced the subsequent risk of hypoglycaemia (Table 1).

### CGM use in prediabetes

An RCT in individuals with prediabetes showed that isCGM combined with lifestyle coaching improved blood glucose levels and reduced carbohydrate intake and body weight [35]. A pilot RCT in 13 individuals with prediabetes or type 2 diabetes suggested that rtCGM may facilitate self-monitoring behaviour and increase exercise adherence accompanied by improvements in health-related QoL [36]. Similarly, a qualitative study in 26 individuals at moderate to high risk of developing type 2 diabetes suggested that using a combination of isCGM and a physical activity monitor may increase self-awareness regarding the impact of lifestyle on short-term health and guide behaviour change [37]. However, the feedback provided by the devices lacked meaning for several individuals, posing barriers to making changes to diet and physical activity levels. Hence, these findings highlight the need for further research to explore potential modifications required to digital health technologies, including CGM, to sustain engagement and behaviour change in individuals with prediabetes.

In summary, high-quality evidence demonstrates that both isCGM and rtCGM deliver glycaemic benefits for people with type 2 diabetes, whether treated with insulin or non-insulin therapy. The available data suggest that the mechanisms for improvements in blood glucose levels in response to CGM may not be directly reacted to therapeutic change, as one might assume. Further studies are required to provide a detailed understanding of the impact of CGM on dietary intake and physical activity, in addition to exploring the potential benefits of CGM in those with type 2 diabetes treated with mixed insulins.

## Continuous subcutaneous insulin infusion in type 2 diabetes

Continuous subcutaneous insulin infusion (CSII), also known as insulin pump therapy, has a clear place in the management of type 1 diabetes [38]. In contrast, the guidelines for using CSII in type 2 diabetes are less consistent [39–41].

The OpT2mise RCT, which included 331 individuals with MDI-treated type 2 diabetes, found that, compared with MDI, CSII resulted in a significant 0.7 pp (7 mmol/mol) HbA_1c_ reduction after 6 months, without increased rates of hypoglycaemia, DKA or hospitalisation [42]. In another RCT, individuals randomised to the CSII arm achieved a significant 0.9 pp (9 mmol/mol) HbA_1c_ reduction compared with 0.3 pp (3 mmol/mol) in the MDI arm. After 6 months, the MDI arm crossed over to CSII and at 12 months the individuals continuing CSII had an additional 0.7 pp (7 mmol/mol) reduction in HbA_1c_ and those switching from MDI to CSII experienced a 0.5 pp (5 mmol/mol) HbA_1c_ reduction [43]. Similarly, the VIVID study demonstrated that, compared with MDI, CSII improved HbA_1c_ without increasing body weight or severe hypoglycaemia [44].

Real-world data suggest that using CSII in type 2 diabetes can be safe and effective for improving blood glucose levels, particularly in those individuals with higher HbA_1c_ levels, and is associated with high user satisfaction [45–47]. In one study, the HbA_1c_ reduction was sustained for 6 years, indicating the potential long-term benefits of CSII therapy for those with type 2 diabetes [46].

Initiating CSII in type 2 diabetes has been associated with improved patient-reported outcomes and user satisfaction [48]. A recent real-world study demonstrated that, compared with MDI, use of a tubeless insulin pump in adults with type 2 diabetes contributed to significant behavioural and psychosocial benefits, including improvements in overall well-being, diabetes distress, hypoglycaemia-related concerns and QoL, as well as greater glycaemic improvement [49]. User satisfaction and improved glycaemic outcomes have also been shown in studies exploring the use of simplified CSII systems with no need for pump programming or detailed education sessions [50, 51].

Overall, CSII is safe and effective in populations with type 2 diabetes, especially in those with an HbA_1c_ significantly above target despite MDI. CSII may also be associated with decreased healthcare costs as a result of lower rates of diabetes-related complications [51–54].

## AID systems in type 2 diabetes

AID systems, also known as closed-loop systems, include ‘hybrid’ closed-loop (HCL) therapies, which require carbohydrate counting and user-initiated, pump-delivered meal boluses, and fully closed-loop systems, which eliminate the need for manual mealtime boluses.

An RCT in 136 individuals with type 2 diabetes showed that, compared with subcutaneous insulin therapy, a fully AID system resulted in a significant 24.3 pp TIR increase and 25.9 pp TAR reduction without increasing hypoglycaemia. User satisfaction was also high in the closed-loop group [55]. Similar results were observed in other RCTs performed in inpatient settings [56, 57].

Randomised trials conducted in outpatient settings also suggest glycaemic benefits of fully closed-loop systems [58–60]. A randomised crossover study in 26 adults with type 2 diabetes compared a fully closed-loop system with standard insulin therapy and a masked glucose sensor (control). The authors demonstrated a significant 15 mmol/mol (1.4 pp) HbA_1c_ reduction and 35.3 pp TIR increase without elevated hypoglycaemia rates following closed-loop therapy compared with control [59].

A recent meta-analysis of seven RCTs assessing the efficacy of fully closed-loop systems compared with conventional insulin therapy in 390 people with type 2 diabetes showed that fully closed-loop systems improved TIR (MD +22.40 pp, 95% CI 12.88, 31.91 pp; *p*<0.01) and reduced TAR (MD −22.67 pp, 95% CI −30.87, −14.46 pp; *p*<0.01) without a significant difference in hypoglycaemia [61].

The literature on HCL therapies in type 2 diabetes is limited [62, 63]. A feasibility trial in 24 adults with type 2 diabetes managed in an outpatient setting found that HCL was associated with a 14 mmol/mol (1.3 pp) HbA_1c_ reduction, 21.9 pp TIR increase, 16.9 pp TAR reduction and 0% of time at glucose <3 mmol/l (<54 mg/dl), without a significant change in total daily insulin dose or body weight [62]. Similarly, a prospective single-arm trial demonstrated a substantial glycaemic improvement (TIR increased by 15 pp) without increased hypoglycaemia in 30 adults with type 2 diabetes using HCL therapy [63].

In summary, small studies suggest that closed-loop systems could be a potential future therapeutic option in type 2 diabetes. More long-term follow-up studies are required to assess their clinical and cost-effectiveness.

## Connected insulin devices in type 2 diabetes

Missed and late insulin injections negatively impact blood glucose levels [64]. Connected insulin devices, including tracking insulin pens, and smart insulin pens and caps, can record and transfer data about insulin doses and timing to smartphone applications, as well as provide reminders to bolus and facilitate insulin dose calculations [65]. These features support decision making and inform counselling strategies for the diabetes care team [65–68].

In a randomised trial that aimed to assess the efficacy of a smart insulin pen cap for the management of individuals with suboptimally controlled type 2 diabetes (intervention group: feedback and alarm notifications; control group: masked device without alarm notifications), compared with the control group (*n*=40), the intervention group (*n*=40) experienced a greater HbA_1c_ reduction (−0.98 pp [–10 mmol/mol] vs −0.72 pp [–7 mmol/mol]; *p*=0.006) and lower blood glucose levels (8.2 ± 1.9 vs 8.7 ± 2.3 mmol/l [147.0 ± 34 vs 157.6 ± 42 mg/dl]; *p*<0.01). The device was also associated with high user satisfaction [69]. In the STYLCONNECT study, people with type 2 diabetes showed a strong interest in using a device that could automate the collection of their insulin data and integrate data from glucose measurement devices [70]. Another study demonstrated that people with type 2 diabetes preferred connected over non-connected insulin pens because of the capability for automated recording of insulin dose and glucose levels [71].

Evidence around the use of connected insulin devices in type 2 diabetes is still in an early phase. However, existing literature suggests that these systems may have the potential to improve plasma glucose and user satisfaction, highlighting the importance of further research in this area [72].

## Special groups

### Early-onset type 2 diabetes

Type 2 diabetes in young people is associated with an excess lifetime risk of vascular complications and premature death [73–76]. Improving HbA_1c_ is crucial to reduce long-term diabetes-related complications and mortality rates [3, 4]. Despite emerging evidence suggesting the glycaemic benefits of technologies such as CGM in older adults with type 2 diabetes [11, 12], research around the use of such systems in young individuals is scarce and limited to small studies [77, 78]. Small pilot studies suggest that rtCGM is acceptable and feasible and associated with significant improvements in QoL and glycaemic outcomes in adolescents and young adults with type 2 diabetes [77, 78]. To date, there are no studies exploring the impact of CSII or closed-loop systems in young people with type 2 diabetes. Further studies assessing the use of technologies in people with early-onset type 2 diabetes are needed to explore the potential benefit of these therapies in this high-risk cohort.

### Pregnancy and type 2 diabetes

Pregnancy complicated by type 2 diabetes is associated with adverse maternal and fetal outcomes [79]. Maternal hyperglycaemia is a major modifiable risk factor for pregnancy outcomes [79], and it seems logical that CGM could improve blood glucose levels and optimise the care of pregnant women with pre-existing diabetes. rtCGM reduces the risk of adverse fetal outcomes in women with type 1 diabetes [80] and may support the management of women with pre-existing diabetes, including the high-risk type 2 diabetes population [81, 82]. Non-randomised studies suggest that isCGM can be useful for improving blood glucose levels in pregnant women with type 2 diabetes and is accurate and well-received [83, 84]. However, RCT-derived data assessing the efficacy of CGM for maternal glucose management and perinatal outcomes in women with type 2 diabetes are currently lacking, while existing studies involve small numbers of individuals [85–87]. The ADA clinical practice recommendations for the management of diabetes in pregnancy state that there are insufficient data to support CGM use in all individuals with type 2 diabetes and that the decision to use CGM should be individualised [88]. NICE guidelines on the management of diabetes in pregnancy indicate that rtCGM should be considered in pregnant women with insulin-treated type 2 diabetes if they have problematic severe hypoglycaemia or unstable blood glucose levels causing concern despite efforts to optimise plasma glucose [89]. Although the International Consensus on Time in Range defines CGM target ranges for people with diabetes, there are currently no internationally agreed goals for pregnant women with type 2 diabetes [88, 90].

Future research should aim to investigate the impact of CGM in pregnant women with type 2 diabetes, assess associations of CGM metrics with pregnancy outcomes and identify the appropriate amount of time spent within defined glucose targets for this population.

### End-stage renal disease and type 2 diabetes

The evidence for using technologies in the type 2 diabetes population with end-stage renal disease on dialysis is scarce. Observational studies suggest that CGM is an accurate and efficient method of monitoring interstitial glucose levels in individuals receiving haemodialysis [91–95]. Data suggest that there is increased glucose variability during dialysis days, which could be an additional risk factor for cardiovascular complications [96, 97]. CGM can capture glucose variations, guide insulin therapy optimisation and improve glucose levels and hypoglycaemia detection in individuals with insulin-treated type 2 diabetes receiving dialysis [98–100]. However, these outcomes should be interpreted with caution as most of the existing studies are observational with short-term follow-up, include small numbers of participants and no control group, and provide very limited evidence on peritoneal dialysis. RCTs and studies with longer follow-up are therefore needed.

A post hoc analysis of an RCT in a type 2 diabetes population undergoing inpatient haemodialysis showed that, compared with subcutaneous insulin therapy, a fully closed-loop system was associated with a significant 37.6% increase in the proportion of time when blood glucose was within the target range (5.6–10.0 mmol/l [100–180 mg/dl]), without increasing hypoglycaemia [101]. Similarly, an RCT in 26 adults with type 2 diabetes requiring dialysis in an outpatient setting showed that a fully AID system significantly increased TIR by 14.6 pp without increased hypoglycaemia compared with standard insulin therapy [58], suggesting that closed-loop systems could be a novel way to achieve safe and effective glucose management in this vulnerable population.

### Older people and type 2 diabetes

The adoption of diabetes technologies in older people remains at an early stage and clinical knowledge is currently modest. Cognitive impairment, multimorbidity and sensory deficits due to increasing age are important challenges in this group [102, 103], while the significance of reducing hypoglycaemia is emphasised in international recommendations [90].

Two RCTs including people with type 2 diabetes on MDI over the age of 60 years found that CGM was associated with a 0.3–0.5 pp (3–5 mmol/mol) HbA_1c_ reduction compared with SMBG [15, 23]. Additional data suggesting that pump therapy may be beneficial in older people with type 2 diabetes on MDI were described in the OpT2mise trial, which included individuals aged up to 75 years [42]. Another RCT demonstrated that, compared with MDI, a fully closed-loop system resulted in a significant 27.4 pp TIR increase, a 27.7 pp TAR reduction and an unchanged TBR of <1% in 30 people with type 2 diabetes (mean age 69.5 years) requiring nursing support at home. There were no episodes of severe hypoglycaemia or ketoacidosis and both participants and caregivers were highly satisfied with the AID system [60].

A recent review from the International Geriatric Diabetes Society described the low uptake of diabetes technologies in older adults because of individual and healthcare system-related barriers [104]. Future studies should aim to explore the efficacy, safety, role, cost implications and potential barriers of using technologies in older people with type 2 diabetes, including those with multimorbidity and cognitive and functional impairment and those living in supervised facilities.

## Cost-effectiveness of technologies in type 2 diabetes

The increasing prevalence of type 2 diabetes globally, particularly in younger individuals who will live longer with their disease and have an increased risk of costly diabetes-related complications, is expected to result in several challenges for healthcare systems and clinicians. Increased rates of emergency department use and hospital admissions due to diabetes-related complications are associated with significant healthcare costs [105]. Hence, using cost-effective technologies, which improve HbA_1c_ and thereby reduce complications, is imperative.

The cost–benefits of CGM in type 2 diabetes have been described previously [106, 107]. A recent retrospective analysis showed that the mean per-patient per-month cost for diabetes-related medical costs in a type 2 diabetes population decreased by US$424 following ≥6 months of rtCGM use. A decrease in hospital admissions was also reported [108]. Other studies have also demonstrated that CGM use in type 2 diabetes is associated with a reduction in diabetes-related admissions, which would imply cost savings for healthcare systems [24, 33]. A base-case analysis showed that long-term isCGM use was cost-effective compared with SMBG in individuals with type 2 diabetes receiving intensive insulin treatment [109]. Similarly, another analysis demonstrated that rtCGM was likely to be cost-effective compared with SMBG in a type 2 diabetes population receiving insulin therapy, with HbA_1c_ reduction and QoL benefit from reduced fingerstick testing being the main drivers of the outcomes observed [110]. Taken together, the available data suggest that CGM is cost-effective, which has led to the inclusion of such systems in guidelines for the management of type 2 diabetes [40, 111].

Evidence suggesting the cost-effectiveness of CSII in type 2 diabetes is scarce. Compared with MDI, CSII was associated with a gain in quality-adjusted life-years ranging between 0.17 and 0.43 and a 15–20% reduction in diabetes-related complication costs, which mitigated the higher mean lifetime costs [53, 54, 112]. Sensitivity analyses showed that insulin pump therapy was most cost-effective in individuals with the highest baseline HbA_1c_, suggesting that CSII may represent a cost-effective therapeutic alternative for MDI-treated type 2 diabetes populations who have HbA_1c_ levels above target [112].

To date, there are no cost-effective analyses of closed-loop systems in type 2 diabetes, and studies comparing the cost-effectiveness of such systems with that of the available glucose-lowering therapies are needed. Lastly, connected insulin devices in this population are potentially cost saving, but further data are required [72].

## Conclusion

People with type 2 diabetes face several challenges in achieving glycaemic targets. Advances in diabetes technologies have provided tools that can facilitate self-management in this high-risk group, especially those on insulin therapy with HbA_1c_ values above target. Further research will indicate the best place within treatment guidelines of newer technologies such as closed-loop therapies, which have shown very promising results at this initial stage.

## Supplementary Information

Below is the link to the electronic supplementary material.

Figure slide (PPTX 238 KB)

## Abbreviations

AID: Automated insulin delivery
CGM: Continuous glucose monitoring
CSII: Continuous subcutaneous insulin infusion
DKA: Diabetic ketoacidosis
HCL: Hybrid closed-loop
isCGM: Intermittently scanned continuous glucose monitoring
MD: Mean difference
MDI: Multiple daily injections
pp: Percentage points
QoL: Quality of life
rtCGM: Real-time continuous glucose monitoring
SMBG: Self-monitoring of blood glucose
TAR: Time above range
TBR: Time below range
TIR: Time in range

## Authors’ relationships and activities

ALL has received support to attend conferences from Eli Lilly and Novo Nordisk and research support from the Association of British Clinical Diabetologists. LL has received research support from Abbott Diabetes Care and Dexcom, participated in advisory groups for Abbott Diabetes Care, Insulet, Dexcom, Medtronic and Roche Diabetes, and received fees for speaking from Sanofi, Insulet, Medtronic and Abbott. EGW has received personal fees from Abbott, AstraZeneca, Dexcom, Eli Lilly, Embecta, Glooko, Insulet, Medtronic, Novo Nordisk, Roche, Sanofi, Sinocare and Ypsomed, research support from the Association of British Clinical Diabetologists, Abbott, Diabetes UK, Embecta, Insulet, Novo Nordisk and Sanofi, and medical writing support from Abbott, Eli Lilly and Embecta, and has participated in consultancy/been an advisory board member for Abbott, Dexcom, Eli Lilly, Embecta, Insulet, Medtronic, Novo Nordisk, Roche and Sanofi. JZML declares that there are no relationships or activities that might bias, or be perceived to bias, this work.
