# Supplementary material for: The use of technology in type 2 diabetes and prediabetes: a narrative review
Source: Diabetologia. 2024 Jun 29;67(10):2059–74. doi: 10.1007/s00125-024-06203-7 (PMC11446986; doi:10.1007/s00125-024-06203-7)
Supplement: Supplementary file 1 — Figure slide (PPTX 238 KB) [file 125_2024_6203_MOESM1_ESM.pptx]

## Slide 1
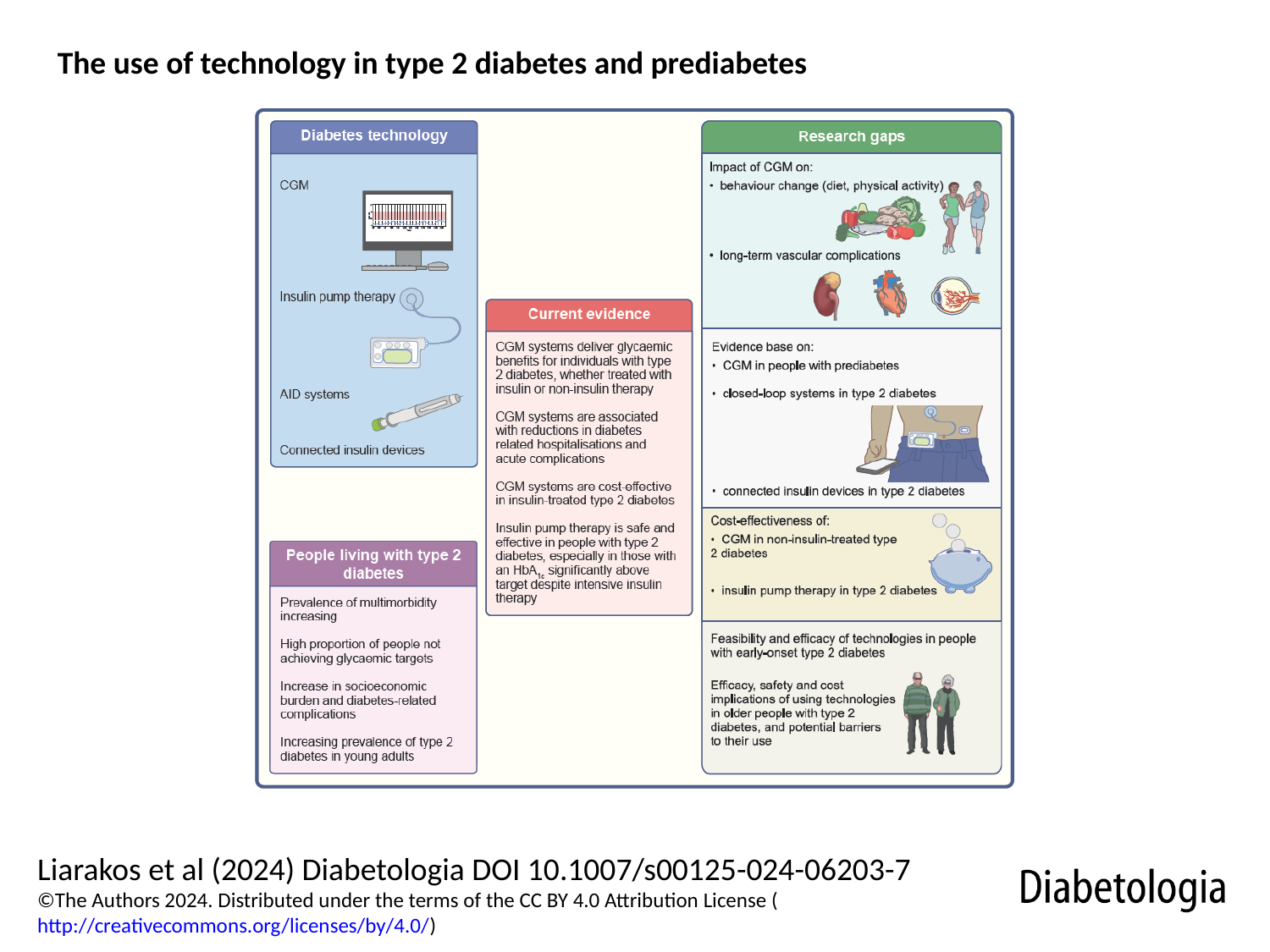

The use of technology in type 2 diabetes and prediabetes
Liarakos et al (2024) Diabetologia DOI 10.1007/s00125-024-06203-7
©The Authors 2024. Distributed under the terms of the CC BY 4.0 Attribution License (http://creativecommons.org/licenses/by/4.0/)
